# Impact of anthocyanin on genetic stability in mammary adenocarcinoma-induced mice treated with methotrexate

**DOI:** 10.1186/s12263-022-00709-8

**Published:** 2022-05-05

**Authors:** Abeer A. Khamis, Rana M. Ibrahim, Gad B. El-hefnawy, Wafaa M. Ibrahim, Ehab M. Ali

**Affiliations:** 1 Biochemistry Division, Chemistry Department, Faculty of Science, Tanta University, Tanta, Egypt; 2 Biochemistry Department, Faculty of Sciences, King Abdulaziz University, Jeddah, Saudi Arabia; 3 Chemistry Department, Faculty of Science, Tanta University, Tanta, Egypt; 4 Department of Medical Biochemistry, Faculty of Medicine, Tanta University, Tanta, Egypt

**Keywords:** Ehrlich’s solid tumor, Antitumor drug, Flavonoids, DNA damage, Poly[adenosine diphosphate (ADP)-ribose] polymerase, Detoxifying enzyme

## Abstract

**Background:**

Genetic instability leads to genome mutations, changes in nucleotide sequences, rearrangements, and gains or losses of part of the chromosomes. This instability can initiate and develop cancer. This study evaluated genomic stability in methotrexate and anthocyanin-treated mammary adenocarcinoma model. Seventy albino mice were divided into seven groups: negative control, anthocyanin, methotrexate, Ehrlich’s solid tumor; Ehrlich’s solid tumor and methotrexate; Ehrlich’s solid tumor and anthocyanin; and Ehrlich’s solid tumor, methotrexate, and anthocyanin groups.

**Results:**

Tumor weight and size were evaluated. Serum arylesterase activity was low in all the induced tumors and those treated with anthocyanin, methotrexate, or both. Poly[adenosine diphosphate (ADP)-ribose] polymerase activity was high, and glutathione S-transferase activity was low in the tumors treated with anthocyanin, methotrexate, or both, compared with that of the untreated tumor. There was an increase in DNA damage in the mice with solid tumors and those injected with methotrexate or methotrexate and anthocyanin, compared with that in the untreated mice.

**Conclusions:**

There was a decrease in genetic instability and DNA damage in the tumor-bearing mice treated with anthocyanin, with a concomitant increase in nuclear poly[adenosine diphosphate (ADP)-ribose] polymerase activity, compared with those of the untreated group. Anthocyanin exerted positive effects in the treatment of mammary adenocarcinoma.

## Introduction

Environmental factors that cause the mutation of key genes (oncogenes, tumor suppressor genes, apoptotic genes, and DNA repair genes) are considered the main cause of cancer [1]. Carcinogens are found in chemicals in food, water, air, and ultraviolet rays emitted from the sun [2]. These factors mutate the key genes that encode vital cell-regulatory proteins. This abnormal cell behavior results in large clumps of abnormal cells that destroy the surrounding normal tissues. Malignant tumors surround tissues, and metastases surround other organs [3]. Genetic instability leads to the onset and development of cancer and key gene mutations that start from modifying the nucleotide sequence to gaining, losing, or rearranging parts of the chromosomes. Owing to the mechanical effect of DNA repair, genetic mutations and spontaneous DNA damage are reduced [4, 5].

Poly[adenosine diphosphate (ADP)-ribose] polymerase (PARP) is an enzyme encoded in humans by the PARP gene. PARP-1 has been related to the regulation of structure and transcription of chromatin, organization of chromosomes, DNA methylation and imprinting, and insulator activity [6]. Moreover, it contributes to DNA repair and programmed cell death that leads to gene stability [7]. Glutathione S-transferases (GSTs) are detoxifying enzymes crucial in catalyzing the conjugation of glutathione (γ-l-glutamyl-l-cysteinyl-glycine) to hydrophobic and electrophilic compounds involving environmental carcinogens, drugs, and oxidative stress products of glutathione via conjugation reactions [8, 9]. In addition, GSTs protect cellular DNA against oxidative stress and numerous toxic molecules that enhance the DNA mutations that promote carcinogenesis. Furthermore, they are involved with leukotrienes and prostaglandin synthesis and modification. GSTs affect the activities of members of the mitogen-activated protein kinase family, modulating signal transduction pathways involved in apoptosis and cell survival [10].

The enzyme, arylesterase, is a member of the paraoxonase family; it is a glycoprotein that is synthesized in hepatocytes and secreted into the blood, where it circulates as high-density lipoprotein (HDL)-associated protein and exhibits a protective effect by detoxifying reactive oxygen species (ROS). It is considered an antioxidant enzyme responsible for protection against lipoprotein oxidation [11].

Methotrexate (MTX) is an antitumor drug and an antifolate with a cytotoxic effect used in the treatment of neoplastic disorders, rheumatoid arthritis, and psoriasis. It limits the speed of cell division and has a strong effect on cancer cells, but it should be administered at high doses to achieve the required therapeutic response [12]. These high doses lead to severe adverse effects, including delayed neurotoxicity, neuropsychological side effects, and leukocytopenia. In addition, it harms hematopoietic stem cells and kidneys [12]. Certain cancer patients develop resistance against MTX treatment, particularly when used in high doses for a long time [13, 14]. The mechanism of its therapeutic efficiency is through changes in folic acid metabolism via the inhibition of dihydrofolate reductase (a key enzyme in this pathway). Folic acid is a water-soluble vitamin involved in purine and pyrimidine syntheses, which are considered essential precursors of the DNA molecule [15].

Natural substances are extremely important in cancer treatment to avoid the toxicity of anticancer drugs and reduce side effects. The plum is a fruit of the subgenus *Prunus* of the genus *Prunus*. The scientific classification of plum is as follows: kingdom, Plantae; order, Rosales; family, *Rosaceae*; subfamily, *Amygdaloideae*; genus, *Prunus*; subgenus, *Prunus*. The plum extract contains polyphenolics, flavonoid compounds, and anthocyanins (ACs) [16]. ACs are a class of water-soluble flavonoids extracted from certain fruits and vegetables, such as plums, red grapes, and purple cabbage [16, 17]. They exhibit a wide range of biological effects, including the prevention of cardiovascular disease, treatment of obesity, and antineoplastic potential. Their antitumor effects are based on a wide range of biological activities, including antioxidant, antiinflammation, differentiation induction, antimutagenesis, inhibition of proliferation by modulating signal transduction pathways, cell cycle arrest induction, and apoptosis stimulation or autophagy of cancer cells, anti-invasion, antimetastasis, and reversing drug resistance in cancer cells and boosting their sensitivity to chemotherapy [18].

Here, the role of ACs in genetic stability and the reduction of the side effects of MTX was evaluated in mice injected with Ehrlich’s solid tumor (EST) as an experimental model for mammary adenocarcinoma.

## Materials and methods

### Extraction of ACs from plums

The plums were purchased from the local Egyptian market. The plum homogenate (50 g/100 mL of distilled water) was prepared, left for 6 h, and centrifuged at 5000 rpm for 15 min. The pH differential method was used to assess the total AC content. The plum extracts were dissolved in 0.2 M potassium chloride solution (pH, 1.0) and 1 M sodium acetate solution (pH, 4.5). The absorbance was measured using a spectrophotometer at 520 and 700 nm in both solutions where ACs were more and less stable, respectively, and the concentration was calculated according to Eq. (1) [19].

1 $$\mathbf{Total}\ \mathbf{A}\mathbf{Cs}\ \mathbf{concentration}\ \left(\mathbf{mg}/\mathbf{L}\right)=\boldsymbol{\Delta}\ \mathbf{A}\times \mathbf{M}\ \mathbf{wt}.\times \mathbf{DF}\times \left[\mathbf{1000}/\boldsymbol{\epsilon}\right],$$

where Δ A = (A_520_ − A_700_)_pH1_ − (A_520_ − A_700_)_pH4.5_, M wt. (g/mol) = molecular weight of cyanidin-3-rutinoside = 595.52, dilution factor (DF) = 25, and **€** (millimolar absorptivity) = 28.800. The plum extract was lyophilized and diluted in distilled water to achieve a final concentration of 10 mg/mL.

### Animals and experimental design

Seventy female albino mice with weights in the range of 20–22 g were donated by the National Institute of Oncology at Cairo University in Egypt. The mice were placed in steel mesh cages and acclimatized in the laboratory for 2 weeks. They were provided a standardized meal and drinking water ad libitum under conventional animal house settings, which included a 12:12-h light-dark cycle and controlled temperature (20–22 °C). The animal care committee of Tanta University’s Faculty of Science in Tanta, Egypt, and the ethics guidelines established in the Guide for the Use and Care of Laboratory Animals were employed in the experiments (IACUC-SCI-TU-0023) [20].

The mice were divided equally into seven groups: **Group I** was the **negative control (NC) group**. **Group II** was the **AC group**. For 4 weeks, in this group, the mice were administered 1 mL of the plum extract containing 10 mg/mL ACs (equal to 500 mg/kg body weight) by oral gavage. The plum extract was lyophilized and diluted in distilled water to achieve a final concentration of 10 mg/mL [21]. **Group III** was the **MTX group**, in which the mice were intraperitoneally injected with MTX on alternate days for 2 weeks at a dose of 2.5 mg/kg body weight [22]. **Group IV** was the **EST group**. Here 1 × 10^6^ Ehrlich’s ascites carcinoma (EAC) cells were subcutaneously implanted in the right thighs of the lower limbs of the mice [22]. **Group V** was the **EST and MTX groups**, in which EAC cells were implanted under the skin of the mice, which were treated with an intraperitoneal (*ip*) injection of MTX on alternate days for two weeks. **Group VI** was the **EST and AC groups**, in which EAC cells were implanted in the mice, which were fed a dose of plum and treated with ACs for 4 weeks. **Group VII** was the **EST, MTX, and AC groups**, in which EAC cells were implanted in the mice, and the mice were injected with MTX and administered a plum dosage. After the study, the mice were euthanized by cervical dislocation under sodium pentobarbital anesthesia (300 mg/kg) intraperitoneally [23]. The blood was immediately collected without an anticoagulant, incubated at 37 °C for 30 min and centrifuged at 3000 ×*g* for 10 min for serum separation. The resulting serum was stored at −80 °C for biochemical analysis. The breast tissues were collected and placed in 10% buffered formalin for histopathological examinations or stored at −80 °C for biochemical analyses. The tumor was excised and sized with a Vernier caliper [24].

### Determination of serum alanine aminotransferase (ALT) and aspartate aminotransferase (AST) activities and creatinine and urea levels

Serum ALT and AST activities were estimated as described in a reported method [25], and urea was determined as [26] creatinine concentrations were assayed using a colorimetric method [27] with commercial kits (Diamond Diagnostics, Egypt).

### Determination of serum arylesterase activity

Arylesterase activity was measured using *p*-nitrophenyl acetate with a reported method [28]. The change in absorbance was recorded at zero time and after 1 min at 405 nm and activity of arylesterase according to Eq. (2).

2 $$\left(\mathrm{nmol}\;p-\mathrm{nitrophenyl}\;\mathrm{acetate}/\min/\mathrm{mL}\right)=\frac{\boldsymbol{\Delta} \boldsymbol{A}\times\boldsymbol{200}\times\boldsymbol{10}^{\boldsymbol{6}}}{\boldsymbol{\epsilon}}$$

where ∆ A = A_1 min_–A_zero_, € = extinction coefficient (17000 M^−1^ cm^−1^), dilution = 200, and 10^6^ = for conversion from M (mmol/mL) to μM (nmol/mL).

### Determination of cytoplasmic GST activity

GST activity was estimated according to Habig et al. [29] by coupling GSH with 1-chloro-2,4-dinitrobenzene (CDNB). First, 100 μL of the sample was placed into a 1-cm quartz cuvette, along with 700 μL of the buffer, 100 μL of CDNB, and 100 μL of GSH. The absorbance was measured after each minute for 3 min at 340 nm [29].

### Membrane, nuclear, and cytoplasmic protein extraction

The membrane, nuclear, and cytoplasmic protein were separated using the Bio Basic Inc. kit (Canada). First, 100 mg of the tumor was weighed and cut into small pieces. Solution A (cytoplasmic protein extraction) was added to extract the cytoplasmic protein after centrifugation. The nuclear proteins were extracted by adding the nuclear protein extraction, solution B (nuclear protein extraction), into the precipitate and centrifuging. The two solutions were combined, and a protease and phosphatase inhibitor cocktail were added to each.

### Determination of nuclear PARP activity

The PARP-1 activity in the cells and tissues was measured by incorporating biotinylated poly(ADP-ribose) (PAR) into histone proteins using Trevigen’s HT Universal PARP-1 Kit purchased from Gaithersburg (MD, USA) [30]. First, 25 μL of a diluted PARP cocktail was added to 25 μL of the nuclear extraction samples and per standard PARP in the well of each plate, which was incubated at room temperature for 1 h. After washing, 50 μL of diluted Strep-HRP and 50 μL of preheated TACS-Sapphire ™ colored substrate were added to each well. Thereafter, the plates were incubated in the dark at room temperature for 15 min, and the reaction was stopped by adding 50 μL of 0.2 M HCl to each well. The absorbance was read at 450 nm using a microplate reader. A standard curve was produced, and the PARP activity was calculated.

### Determination of DNA damage and extraction in tumor tissue

The amount of DNA damage was measured by the diphenylamine (DPA) colorimetric assay [31]. DNA extraction was performed using a GF-1 tissue DNA extraction kit (Vivantis, Malaysia). The genomic DNA was filtered with low-salt solutions, and the ratio of A260/280 DNA extracted using nano drops was measured and fell between 1.7 and 1.9 [32].

### Evaluation of genomic instability by random-amplified polymorphic DNA (RAPD) analysis

The genetic instability of the eluted DNA from the different groups was evaluated by RAPD analysis. Six short primers were used in the RAPD assay [33, 34], and a polymerase chain reaction (PCR) was performed with a PCR Master Mix. Maximo Taq DNA polymerase 2X-preMix was supplied from Gene ON (Germany) according to a reported method [35, 36]. The RAPD was formed in different regions of the genome.

Lyophilized primers were supplied from Vivantis (Malaysia). The lyophilized primers were reconstituted by the addition of sterile water to a final concentration of 100 picomoles/μL, distributed in aliquots, and stored at −20 °C. Each tube contained 25 μL Taq polymerase, 0.5 μL primer, and 60 μL DNA and was completed to 50 μL using distilled water. The tubes were transferred to a thermal cycler (Whatman, Biometra, Germany) where they were subjected to a cycle of initial activation at 94 °C for 4 min and 40 cycles (1 min at 94 °C for denaturation and 1 min for primer annealing). The temperature of annealing was 34 °C for the OPA-06, OPB-05, and OPU-16 primers and 32 °C for the OPA-07, OPA-8, and OPB-18 primers. The primer extension was 2 min at 72 °C, and the final extension cycle was 10 min at 72°C.

**Table:** 

	**Primer sequences (5′–3′)**	**Melting temperature (Tm)**	**GC content (%)**
**OPA-06**	5′ GGTCCCTGAC 3′	41°C	70
**OPA-07**	5′ GAAACGGGTG 3′	36.9°C	60
**OPA-08**	5′ GTGATCGCAG 3′	36.9°C	60
**OPB-05**	5′ TGCGCCCTTC 3′	41°C	70
**OPB-18**	5′ CCACAGCAGT 3′	36.9°C	60
**OPU-16**	5′ CTGCGCTGGA 3′	41°C	70

**Genomic template stability (GTS%)** is a qualitative measure reflecting changes in the RAPD profiles according to Eq. (3). GTS% was calculated as follows:

3 $$\mathbf{100}\times \left(\mathbf{1}-\mathbf{a}/\mathbf{n}\right),$$

where *a* was the RAPD profile detected in each treated sample and *n* was the total number of bands in the control according to Eq. (4) [33, 34].

4 $$\mathbf{Genomic}\ \mathbf{instability}\ \left(\%\right)=100-\mathrm{GTS}\%,$$

A monomorphic band is a band of DNA present in all individuals, while a polymorphic band is a band present in an individual or certain individuals but absent in another individual [37].

### Histopathological examination of tumor tissue

The tissues were placed in paraffin, stained with Harris Hematoxylin, and then stained with eosin. The stained slides were observed with high-power field microscopy. The necrotic area was qualitatively determined regarding the neoplastic cells [38].

### Statistical analysis

The obtained data were analyzed using the GraphPad InStat (version 3.05). The values are expressed as triplicates ± SD, and probability values of *p* ≤ 0.05 were significant. The data were evaluated by one-way analysis of variance to determine the statistical significance of the differences among groups using Duncan’s multiple range test.

## Results

### Tumor volume and weight

The percentages of changes in the tumor volume and weight of groups V, VI, and VII significantly decreased by 40 and 77%, 30 and 58%, and 65 and 78%, respectively, compared with those of group IV (Table 1).

**Table 1:** Tumor volume (cm^3^) and tumor weight (g) in groups IV, V, VI, and VII

Groups	Tumor volume (cm^**3**^)	Tumor weight (g)
**G IV: EST**		
**Range**	0.83-5.1	0.13-2.74
**Mean±SD**	2±0.45	1.12±0.14
**G V: EST & MTX**		
**Range**	0.15-2.94	0.05-0.45
**Mean± SD**	1.2±0.34	0.26±0.018
*p*^*1*^	< 0.01 ↓	<0.001 ↓
**% change**	-40%	-77%
**G VI: EST & ACs**		
**Range**	0.28-3.2	0.1-0.89
**Mean± SD**	1.4±0.26 ↓	0.47±0.035
*p*^*1*^	< 0.01	<0.001 ↓
*p*^*2*^	N.S	<0.001 ↑
**% change**	-30%	-58%
**G VII: EST & MTX & ACs**		
**Range**	0.15-1.88	0.11-0.40
**Mean± SD**	0.7±0.09	0.25±0.01
*p*^*1*^	< 0.001 ↓	<0.001 ↓
*p*^*2*^	< 0.01 ↓	N.S.
*p*^*3*^	< 0.01 ↓	<0.01 ↓
**% change**	−65%	−78%

*p*^*1*^ versus EST, *p*^*2*^ versus EST and MTX , *p*^*3*^ versus EST and ACs, Significant *p* < 0.05, *NS* not significant

### Serum ALT and AST activities and creatinine and urea levels

The percentage of changes in the serum ALT activity of group VII was 43.87%, compared with that of group I. There was a significant increase at p < 0.001 in the serum ALT activity in group V, compared with that of group I. The serum AST activity in group VII showed a slightly significant increase, compared with that of the NC group. However, for group II, the serum AST activity significantly decreased at p < 0.001, and the percentage of change decreased by 83.4%, compared with that of group I (Table 2).

**Table 2:** Determination of serum ALT and AST activities (unit/mL), creatinine and urea levels (mg/dL), and arylesterase activity (nmol/min/mL) in the different groups

Groups	ALT Unit/ml	AST Unit/ml	Creatinine mg/dl	Urea mg/dl	Arylesterase nmole/min/ml
**G I: NC**					
**Range**	37–42	63–75	0.49–0.59	38–42	494.12–528.82
**Mean± SD**	39.2±1.9	68.4±4.7	0.54±0.04	40±1.6	522.79±17.68
**G II: ACs**					
**Range**	34–39	22–34	0.41–0.57	30–39	468.24–582.35
**Mean± SD**	36.6±2.1	28±4.7	0.5±0.07	35.2±3.6	530.26±43.04
*p*^*1*^	N.S.	↓< 0.01	N.S.	N.S.	N.S.
**% of change**	–6.6%	–83.4%	–7.4%	–12%	1.43%
**G III: MTX**					
**Range**	215–265	224–296	0.60–0.98	55–65	210.59–258.82
**Mean± SD**	244.4±22.3	252.4±29.4	0.79±0.18	59±3.9	239.41±18.76
*p*^*1*^	↑< 0.001	↑< 0.001	N.S.	↑< 0.001	↓< 0.001
**% of change**	523.5%	49.88%	46.29%	47.5%	-59.72%
**G IV: EST**					
**Range**	49–54	600–676	0.55–0.67	86–90	120–135
**Mean± SD**	51.6±2.1	628.2±29.6↑<	0.6±0.05	88±1.6	127.6±5.94
*p*^*1*^	N.S.	0.001	N.S.	↑< 0.001	↓< 0.001
**% of change**	31.6%	273%	11.11%	120%	-75.59%
**G V: EST & MTX**					
**Range**	48–57	919–980	0.86–1.98	48–57	262.35–290.58
**Mean± SD**	52.2±3.8	945.6±24.1	1.34±0.47	51.2±3.6	274.99±10.17
*p*^*1*^	N.S.	↑< 0.001	↑< 0.001	↑< 0.001	↓< 0.001
*p*^*2*^	N.S.	↑< 0.001	↓< 0.001	↓< 0.01	↑< 0.001
**% of change**	33.1%	461.5%	148.15%	28%	-47.39%
**GVI: EST & ACs**					
**Range**	56–65	712–853	0.55–0.60	35–47	254.12–289.41
**Mean ±SD**	61.6±3.6	810.6±56.2	0.57±0.02	41.8±4.5	270.79±12.88
*p*^*1*^	↑< 0.01	↑< 0.001	N.S.	N.S.	↓< 0.001
*p*^*2*^	N.S.	↑< 0.001	N.S.	↑< 0.001	↑< 0.001
*p*^*3*^	N.S.	↑< 0.001	< 0.001	↓ < 0.01	N.S.
**% of change**	57.14%	381.4%	5.5%	4.5%	–48.20%
**GVII: EST & MTX & ACs**					
**Range**	55–59	835–980	0.24–0.36	40–49	231.76–288.24
**Mean± SD**	56.4±2.1	878.6±60.9	0.3±0.05	43.8±3.9	277.08±28.1
*p*^*1*^	N.S.	↑< 0.001	N.S.	N.S.	↓< 0.001
*p*^*2*^	N.S.	↑< 0.001	N.S.	↑< 0.001	↑< 0.001
*p*^*3*^	N.S.	N.S.	↓< 0.01	↓< 0.05	N.S.
*p*^*4*^	N.S.	N.S.	N.S.	N.S.	N.S.
**% of change**	43.87%	421.7%	-44.44%	9.5%	-46.99%

*P*^*1*^ versus negative control, *P*^*2*^ versus EST, *P*^*3*^ versus EST and MTX, *P*^*4*^ versus EST and ACs, Significant *p* < 0.05, *NS* not significant

The serum creatinine level showed a highly significant increase at p < 0.001 in group V, compared with those of groups I and IV. The serum urea level showed a highly significant increase at p < 0.001 in groups III and IV, compared with that of group I (Table 2).

### Serum arylesterase activity

The percentages of change in the serum arylesterase activity decreased by 59.72, 75.59, 47.39, 48.20, and 46.99% in groups III, IV, V, VI, and VII, respectively, compared with that of group I. The serum arylesterase activity showed a significant increase at p < 0.001 in groups V, VI, and VII, compared with that of group IV (Table 2).

### Cytoplasmic GST activity

The percentages of change in the cytoplasmic GST activity decreased by 61.48, 43.81, and 53.99% in groups V, VI, and VII, respectively, compared with that of group IV (Table 3).

**Table 3:** Cytoplasmic (GST) (nmol/min/mg protein) activity and fragmented DNA in groups IV, V, VI, and VII

Groups	GST nmol/min/mg protein	PARP Unit/mg protein	Fragmented DNA %
**G IV: EST**			
**Range**	47.5-53.70	0.01-0.06	17.5-27.86
**Mean±SD**	50.1±2.6	0.04±0.02	23.09±3.2
**G V: EST & MTX**			
**Range**	12.24-28.33	0.04-0.11	26.17-33.52
**Mean± SD**	19.3±5.8	0.08±0.03	28.59±2.3
*P*^*1*^	↓< 0.001	N.S.	N.S.
**% of change**	-61.48%	100%	23.82%
**GVI: EST & ACs**			
**Range**	25.07–30.15	0.07–0.14	16.99–28.45
**Mean±S.D**	28.15±1.9	0.09±0.03	24.08±4.3
*P*^*1*^	↓< 0.001	↑< 0.05	N.S.
*P*^*2*^	↑< 0.01	N.S.	N.S.
**% of change**	-43.81%	125%	4.3%
**GVII: EST & MTX & ACs**			
**Range**	20.00-24.91	0.15-0.19	20.66-39.92
**Mean±S.D**	23.05±2.4	0.17±0.02	30.55±6.7
*P*^*1*^	↓< 0.001	↑< 0.001	↑< 0.05
*P*^*2*^	N.S.	↑< 0.001	N.S.
*P*^*3*^	N.S.	↑< 0.001	↑< 0.05
**% of change**	-53.99%	325%	32.3%

*P*^*1*^ versus EST, *P*^*2*^ versus EST and MTX, *P*^*3*^ versus EST and ACs, Significant *p* < 0.05 , *NS* not significant

### Nuclear PARP activity in the nuclear extraction of tumor tissue

**Fig. 1:**
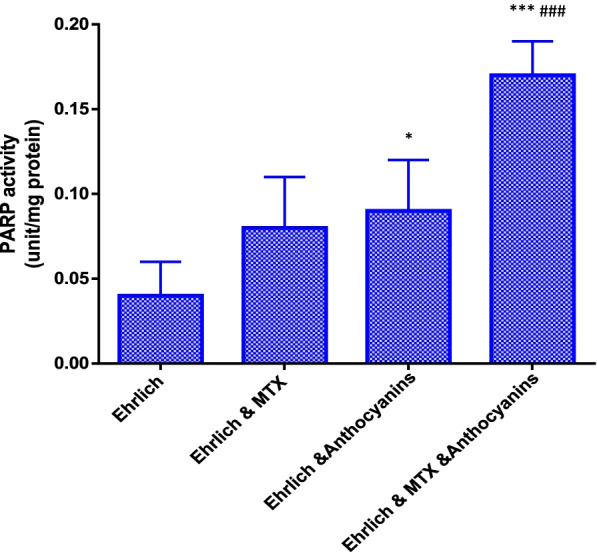
Poly(ADP-ribose) polymerase (PARP) activity in the different studied groups, where *p < 0.05 versus group IV; ***p < 0.001 versus group IV; ###*p* < 0.001 versus group V

The PARP activity in group VI showed a significant increase, compared with that of group IV. Additionally, the PARP activity in group VII showed a highly significant increase, compared with those of groups IV, V, and VI (Fig. 1). The percentages of change in the PARP activity increased by 100, 125, and 325% in groups V, VI, and VII, respectively, compared with that of group IV (Table 3).

### DNA damage in tumor tissue

The percentages of change in the fragmented DNA increased by 23.82, 4.3, and 32.3% in groups V, VI, and VII, respectively, compared with that of group IV (Table 3).

### Genomic instability by RAPD analysis

**Fig. 2:**
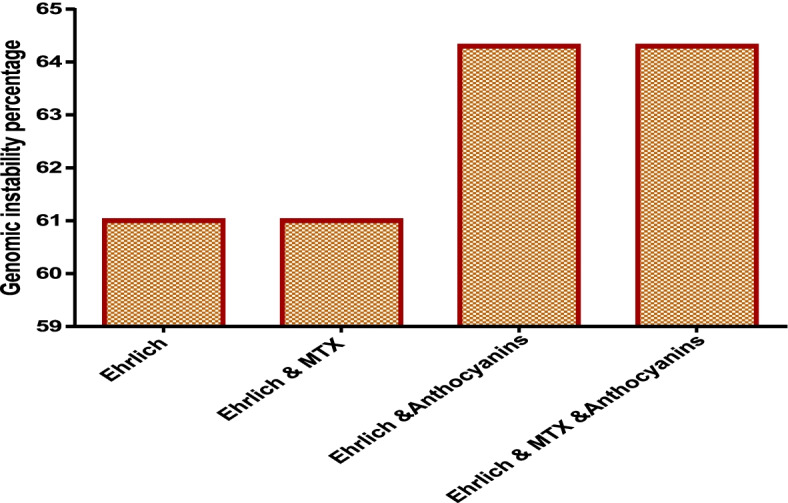
Genomic instability % of the different studied groups

**Fig. 3:**
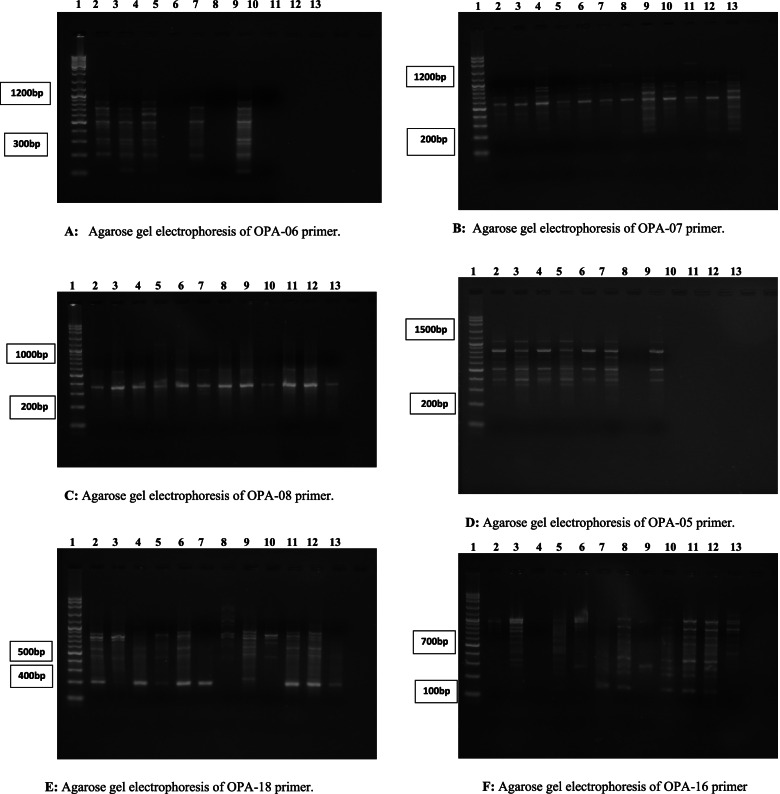
**A** Agarose gel electrophoresis of OPA-06 primer in tumor mice. **B** Agarose gel electrophoresis of OPA-07 primer in tumor mice. **C** Agarose gel electrophoresis of OPA-08 primer in tumor mice. **D** Agarose gel electrophoresis of OPB-05 primer in tumor mice. **E** Agarose gel electrophoresis of OPB-18 primer in tumor mice. **F** Agarose gel electrophoresis of OPU-16 primer in tumor mice. Random-amplified polymorphic DNA (RAPD) profile generated by primers (lane 1 represents the DNA ladder, lanes 2–4 represent group IV, lanes 5–7 represent group V, lanes 8–10 represent group VI, and lanes 11–13 represent group VII)

The changes in the RAPD profiles included a variation in the band intensity, loss of normal bands, and appearance of a new band. Polymorphism observed in the RAPD profiles included the disappearance of the normal band and the appearance of new bands, compared with that in the control profiles. The average was calculated for each experimental group. Although the GTS of group I was 100%, those of groups IV, V, VI, and VII were 39, 39, 35.5, and 35.5%, respectively. The genomic instability was 61% for groups IV and V and 64.3% for groups VI and VII (Table 4, Figs. 2 and 3).

**Table 4:** Genomic instability of the studied groups

	Group IV	Group V	Group VI	Group VII
**GTS%**	39%	39%	35.5%	35.5%
**Genomic instability%**	61%	61%	64.3%	64.3%

### Histopathological examination of tumor tissue

The tumor section stained using hematoxylin and eosin (H&E) in group IV showed large sheets of malignant tumor cells infiltrating the muscle in this group with mild areas of necrosis (+) in four out of ten studied animals. In addition, the stained sections in group V showed sheets of malignant tumor cells with moderate necrosis (++) of the tumor tissue in five out of ten studied animals and extensive necrosis (+++) in three animals, while two animals showed diffuse necrosis of the tumor tissue (++++).

**Fig. 4:**
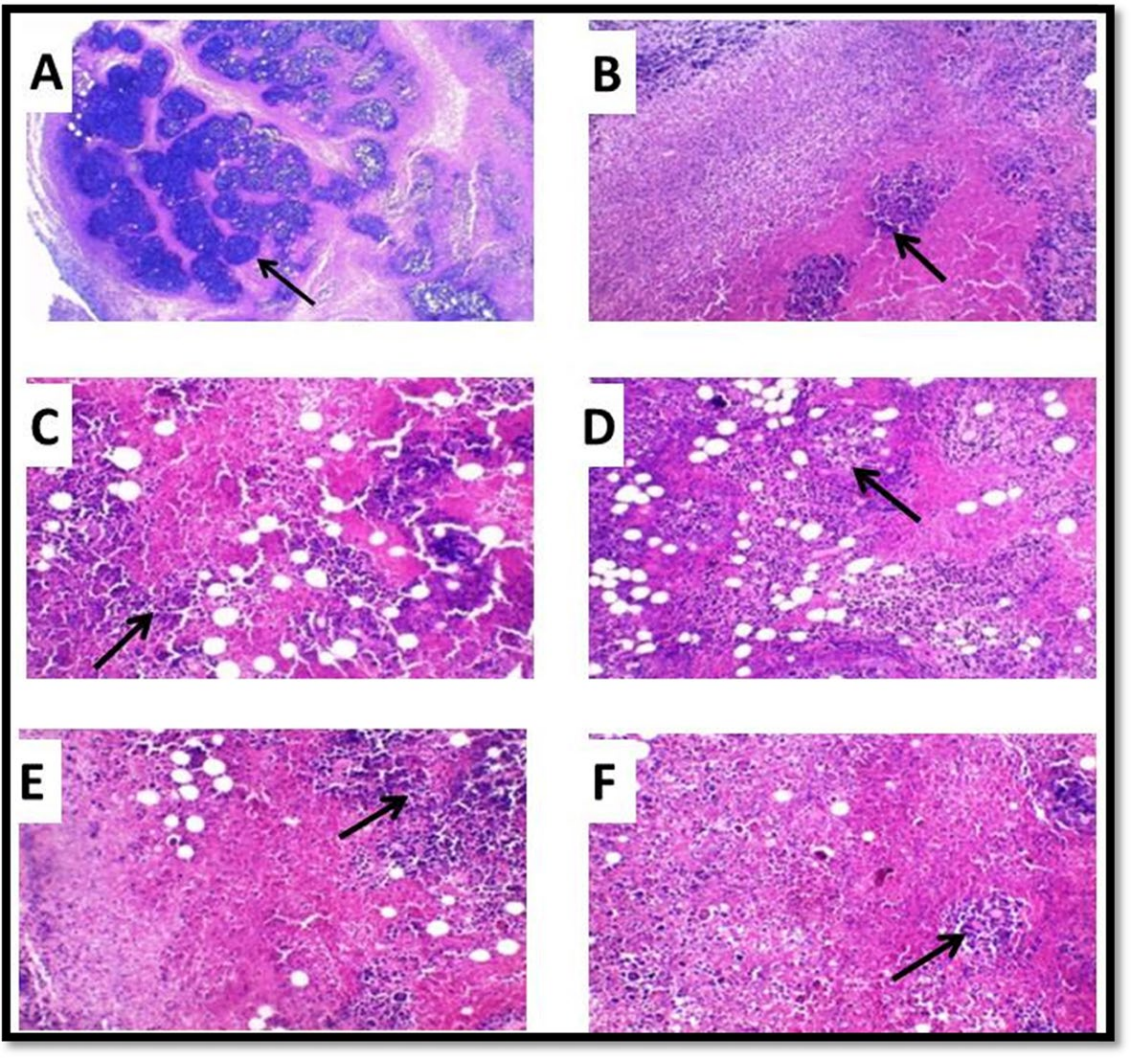
Photomicrograph of tumor tissue. **A** Malignant cells with minimal areas of necrosis in group IV (hematoxylin and eosin (H&E), ×100). **B** Malignant cells with moderate areas of necrosis in group V (H&E, ×200). **C** Malignant cells with extensive areas of necrosis in group V (H&E, ×200). **D** Malignant cells with moderate areas of necrosis in group VI (H&E, ×200). **E** Malignant cells with extensive areas of necrosis in group VI (H&E ×200). **F** Malignant cells with diffuse areas of necrosis in group VII (H&E ×200)

The stained sections from the tumor in group VI showed sheets of malignant tumor cells with moderate necrosis (++) of the tumor tissue in three out of ten studied animals and extensive necrosis (+++) in five animals, while two animals showed diffuse necrosis of the tumor tissue (++++). Additionally, the stained sections in group VII showed sheets of malignant tumor cells with extensive necrosis (+++) of tumor tissue in six out of ten studied animals, while four animals showed diffuse necrosis of the tumor tissue (++++) (Fig. 4).

### Correlation between PARP, GST, and fragmented DNA

**Fig. 5:**
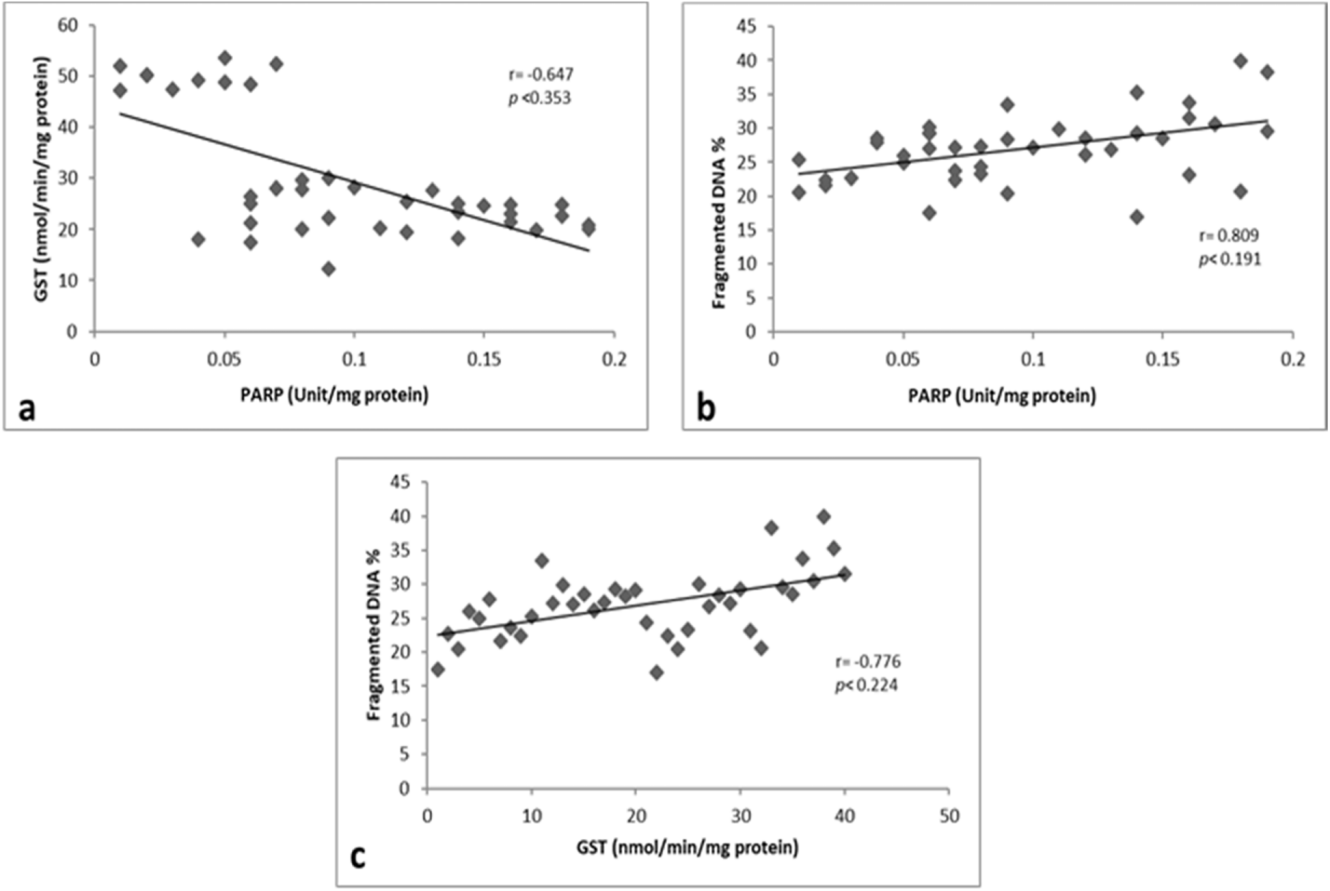
Correlations between **A** PARP and GST activities, **B** PARP activity and fragmented DNA, and **C** GST activity and fragmented DNA

A negative correlation existed between the nuclear PARP and cytoplasmic GST activities (Fig. 5a). Additionally, there was a negative correlation between the cytoplasmic GST activity and fragmented DNA (Fig. 5c). However, a positive correlation was observed between the nuclear PARP activity and fragmented DNA (Fig. 5b).

## Discussion

This study evaluated the genomic instability in the pathogenesis of an experimental model of mammary adenocarcinoma and assessed the antitumor potential of ACs. A decrease in the tumor volume of the treated groups was observed after the experiment. The results correlated with a previous study [39] where there was a decrease in the tumor size and body weight of mice. Statistically, a decrease in the tumor weight of the AC-treated groups, compared with that of the EST-bearing groups, has been recorded [40]. Correlating with the present results, Huang et al. [41] observed that mulberry ACs (MACs) could reduce tumor growth and volume in mice. In addition, ACs have been shown to inhibit malignant cell growth, stimulate apoptosis, and modulate oncogenic signaling events [42, 43]. The *ip* administration of MTX and/or atorvastatin to mice significantly decreased tumor volume, compared with the group implanted with EACs into the right thighs of their hind limbs.

The decrease in tumor volume was significant in a group that received a combination of MTX/atorvastatin, compared with that of the group that received either MTX or atorvastatin [44, 45]. PARP-1 is a nuclear multifunctional enzyme that is important in the cellular response to oxidative stress; oxygen radicals damage DNA strands and activate PARP-1 that repairs DNA, but excessive PARP-1 activation leads to cell death through energy depletion [46, 47]. PARP-1 activation is an extremely sensitive indicator of DNA damage, appearing considerably early and exceeding in magnitude the augmentation of DNA nicks monitored by terminal-deoxynucleotidyltransferase [48].

Here, we assessed the activity of the PARP-1 enzyme with ACs. Hageman et al. [49] hypothesized that oxidative stress induces chronic and systemic PARP-1 activation and increases NAD^+^ turnover. They assumed that under chronic oxidative stress, cellular ATP production, and resynthesis of NAD^+^ may be inadequate.

In the long term, these conditions may lead to reduced NAD^+^ status [49]. The consequence of reduced cellular NAD^+^ is an impaired generation of high-energy phosphates, resulting in the progressive loss of mitochondrial membrane potential, oxidative phosphorylation failure, and ATP depletion [50]. When bound to the DNA adduct, the enzyme starts to ribosylate itself, leading to the recruitment of repair systems and stimulation of phosphorylation and stabilization of p53 [51].

Therefore, DNA damage leads to the activation of PARP to promote DNA repair, which explains why PARP-1 activity increased in groups V and VI, compared with that in group IV. We also observed that PARP activity in group VII significantly increased, compared with those of groups IV, V, and VI because of the AC deactivation of PARP to repair the damage. These data correlated with other studies on cancer-protective effects of cyanidin glucosides and have been demonstrated in studies using cancer cell lines including apoptotic effects via G2/M growth cycle arrest [52].

Furthermore, ACs and anthocyanidins, which are extracted from berries and grapes, have shown pro-apoptotic effects in multiple cell types in vitro studies [53, 54]. Cell cycle arrest and apoptosis have been clarified in mutated cells exposed to cherry ACs [55, 56] and in human oral cancer cell lines treated with cranberry extracts [57].

Many studies have reported that ACs show a wide range of biological activities [58] including antioxidant [59], anti-inflammatory [60], antimicrobial [61], anticarcinogenic and pro-apoptotic [62], vision improvement [63], and neuroprotective effects [64]. Moreover, ACs exhibit a variety of effects on the blood vessels [65] and platelets [66] that may decrease the danger of coronary heart disease. The anticarcinogenic activities of ACs in the initial stage of tumorigenesis inhibit the invasion and metastasis of the tumor [18].

GSTs are key cellular proteins that protect against electrophilic toxins and are involved in physiologically important functions; it is an antioxidant enzyme [67]. The results of this study indicated that cytoplasmic GST activity significantly decreased in group V, compared with that in group IV because of the generation of ROS, which exhausted this enzyme, thereby reflecting the adverse effect of MTX on this balanced antioxidant system. GST activity significantly decreased in groups V, VI, and VII, compared with that in group IV. In addition, the percentages of change in the cytoplasmic GST activity of groups V, VI, and VII decreased by 61.48%, 43.81%, and 53.99%, respectively, compared to that in group IV [68]. Our data indicated that there was a highly significant decrease in arylesterase activity in groups VII and IV, compared with that in group I. Additionally, there was a highly significant decrease in the arylesterase activity in groups III, II, and VII, compared to that in group I. In addition, the percentages of change in the arylesterase activity for groups II–VII were 1.43%, −59.72%, −75.59%, −47.39%, −48.20%, and −46.99%, compared to that in group I. This finding may be due to the ameliorative effect of AC supplementation in the decrease of oxidative stress and the enhancement of antioxidant enzyme activities [68].

The differences in the DNA damage response between normal and malignant cells regularly underlie the utility of DNA damaging agents in cancer treatment [69]. The results of the present study indicated that there was an increase in DNA damage in groups V and VII, compared with that in group IV. There was an insignificant increase in the DNA damage in group VI, compared with that in group IV and a decrease in the DNA damage in group VI, compared with those in groups V and VII. These results correlated with a previous study [70] that showed the role of MTX in the induction of DNA damage.

In addition, another study [71] confirmed that MTX stimulated double-strand breaks. The decrease in DNA damage in group VI, compared with that in group V was due to the protective role of ACs. This result correlated with another study [72] that indicated the antioxidant activity of ACs and its role in protecting DNA from damage. Regarding the assessment of genomic instability, which is a driving force of tumorigenesis [73], numerous types of molecular markers are available for the assessment of the extent of genetic variation. These comprise RAPD, restriction fragment length polymorphism, and amplified fragment length polymorphism. RAPD markers are increasingly being used in genetic research, owing to their speedy process and simplicity. This technique allows the analysis of genomic variation without previous knowledge of DNA sequences [74].

Here, the RAPD profiles showed substantial differences among the studied groups with changes in the number and size of amplified DNA fragments for different primers. The genomic instability percentage was 61% in groups IV and V. This percentage increased to 64.3% in groups VI and VII. It was reported that an alteration in band patterns detected in DNA fingerprint analyses reflected DNA alterations from single-base changes (point mutations) to complex chromosomal rearrangements [75].

Our data confirmed that a positive correlation existed between nuclear PARP activity and fragmented DNA. This result may be explained by a previous study [76] that showed that DNA strand break scan induced excessive activation of PARP-1 activity, leading to the ADP-ribosylation of nuclear proteins as the initiation of DNA repair and the ADP-ribosylation of endonucleases to prevent further DNA damage [77]. Although moderate PARP-1 activation is a critical step in DNA repair, excessive PARP-1 activation depletes cellular NAD1 and ATP content, thereby causing oncotic necrosis [78, 79].

## Conclusion

Anthocyanins have been reported to exert positive effects in the treatment of mammary adenocarcinoma. The possible mechanism of its antitumor activity is by increasing DNA damage driving many cell death pathways, increasing PARP activity, and decreasing GST activity, although it does not affect genomic instability. In addition, AC treatment ameliorates the side effect of MTX.

## Data Availability

All data generated or analyzed during this study are included in this published article.
